# Comparison of an Ensemble of Machine Learning Models and the BERT Language Model for Analysis of Text Descriptions of Brain CT Reports to Determine the Presence of Intracranial Hemorrhage

**DOI:** 10.17691/stm2024.16.1.03

**Published:** 2024-02-28

**Authors:** A.N. Khoruzhaya, D.V. Kozlov, K.M. Arzamasov, E.I. Kremneva

**Affiliations:** Junior Researcher, Department of Innovative Technologies; Scientific and Practical Clinical Center for Diagnostics and Telemedicine Technologies of the Moscow Department of Health, Bldg 1, 24 Petrovka St., Moscow, 127051, Russia; Junior Researcher, Department of Medical Informatics, Radiomics and Radiogenomics; Scientific and Practical Clinical Center for Diagnostics and Telemedicine Technologies of the Moscow Department of Health, Bldg 1, 24 Petrovka St., Moscow, 127051, Russia; Head of the Department of Medical Informatics, Radiomics and Radiogenomics; Scientific and Practical Clinical Center for Diagnostics and Telemedicine Technologies of the Moscow Department of Health, Bldg 1, 24 Petrovka St., Moscow, 127051, Russia; Leading Researcher, Department of Innovative Technologies; Scientific and Practical Clinical Center for Diagnostics and Telemedicine Technologies of the Moscow Department of Health, Bldg 1, 24 Petrovka St., Moscow, 127051, Russia; Senior Researcher; Research Center for Neurology, 80 Volokolamskoye Shosse, Moscow, 125367, Russia

**Keywords:** computed tomography, diagnostic reports, intracranial hemorrhage, natural language processing, machine learning, BERT

## Abstract

**The aim of this study** is to train and test an ensemble of machine learning models, as well as to compare its performance with the BERT language model pre-trained on medical data to perform simple binary classification, i.e., determine the presence/absence of the signs of intracranial hemorrhage (ICH) in brain CT reports.

**Materials and Methods:**

Seven machine learning algorithms and three text vectorization techniques were selected as models to solve the binary classification problem. These models were trained on textual data represented by 3980 brain CT reports from 56 inpatient medical facilities in Moscow. The study utilized three text vectorization techniques: bag of words, TF-IDF, and word2vec. The resulting data were then processed by the following machine learning algorithms: decision tree, random forest, logistic regression, nearest neighbors, support vector machines, Catboost, and XGboost. Data analysis and pre-processing were performed using NLTK (Natural Language Toolkit, version 3.6.5), libraries for character-based and statistical processing of natural language, and Scikit-learn (version 0.24.2), a library for machine learning containing tools to tackle classification challenges. MedRuBertTiny2 was taken as a BERT transformer model pre-trained on medical data.

**Results:**

Based on the training and testing outcomes from seven machine learning algorithms, the authors selected three algorithms that yielded the highest metrics (i.e. sensitivity and specificity): CatBoost, logistic regression, and nearest neighbors. The highest metrics were achieved by the bag of words technique. These algorithms were assembled into an ensemble using the stacking technique. The sensitivity and specificity for the validation dataset separated from the original sample were 0.93 and 0.90, respectively. Next, the ensemble and the BERT model were trained on an independent dataset containing 9393 textual radiology reports also divided into training and test sets. Once the ensemble was tested on this dataset, the resulting sensitivity and specificity were 0.92 and 0.90, respectively. The BERT model tested on these data demonstrated a sensitivity of 0.97 and a specificity of 0.90.

**Conclusion:**

When analyzing textual reports of brain CT scans with signs of intracranial hemorrhage, the trained ensemble demonstrated high accuracy metrics. Still, manual quality control of the results is required during its application. The pre-trained BERT transformer model, additionally trained on diagnostic textual reports, demonstrated higher accuracy metrics (p<0.05). The results show promise in terms of finding specific values for both binary classification task and in-depth analysis of unstructured medical information.

## Introduction

Application of various machine learning algorithms in qualitative analysis of clinical data becomes increasingly important in scientific research and healthcare practice. The scope of text information increases annually and creates difficulties for the following persons: medical professionals collecting and statistically processing medical data; researchers analyzing this data to obtain new scientific knowledge; software developers [1, 2].

Unstructured texts, such as medical records, diagnostic records, patient reviews, and comments on social networks, are a rich source of data for scientific research. However, manual analysis of such texts is time-consuming and is associated with errors. It is especially important to quickly and efficiently extract the required information from X-ray reports. This information and its subsequent automatic processing can facilitate effective decision-making within the shortest time when diagnosing a particular pathology, which is critical in case of emergency and urgent medical care — for example, for the intracranial hemorrhage (ICH) diagnosis [3, 4].

Various natural language processing (NLP) techniques are used to convert written text into machine-treatable datasets [5]. Such data can be analyzed using machine learning (ML) models [6], including advanced approaches that involve deep learning (DL). Deep learning is a subset of machine learning techniques that uses man-made neural networks to analyze data. DL algorithms can be applied to analyze medical texts and identify data patterns and relationships. However, each algorithm has its disadvantages and is not very accurate, for example, with low-structured texts [7]. Thus, it is recommended to create ensembles of algorithms that combine the best features of all specific models.

The effectiveness of ensembles of machine learning algorithms for NLP issues in medicine was demonstrated in a limited number of publications on analysis of medical information and extraction of specific features from the text [8, 9]. However, the available data indicate that this approach can be applied to binary or multi-class classification with fairly high accuracy, which is higher than that of a single algorithm. For example, the AUROC indicator for the “intracranial mass effect” feature in text records of brain CT was 0.96 for the ensemble XGBoost model with the TF-IDF (term frequency-inverse document frequency) technique used for text vectorization [8].

Recently, the Bidirectional Encoder Representations from Transformers (BERT) model has been used for natural language processing tasks including low-structured medical texts with high variability in descriptions [10]. The BERT language model can solve many natural language processing problems due to the fact that it reads text data both from right to left and from left to right (bidirectionally). Therefore, it demonstrates better results compared to its predecessors, which were one-directional. BERT consists of several layers that form a “transformer”, which studies contextual relationships and proximity between different words in the text data. Transformers focus on word analysis: they link words to recognize the semantics of a sentence to better understand its overall meaning [11]. Even with no additional training on specific medical texts, the BERT model can achieve fairly high accuracy values due to its preliminary training on big data with other purposes (for example, for image analysis); subject to additional training, BERT can even surpass other existing methods of automatic text processing [12, 13].

**The aim of this study** is to create, train and test an ensemble of machine learning models that is capable of achieving maximum accuracy, as well as to compare its performance with the BERT language model pre-trained on medical data to perform simple binary classification, i.e., determine the presence/absence of the signs of intracranial hemorrhage in brain CT reports.

## Materials and Methods

The input data is the data download from the Unified Radiological Information Service of the Unified Medical Information Analysis System (URIS UMIAS) [14], containing 34,188 records of examinations as a result of non-contrast brain CT in 56 medical organizations of inpatient medical care. Data analysis and pre-processing were performed by using NLTK (Natural Language Toolkit, version 3.6.5), libraries for character-based and statistical processing of natural language, and Scikit-learn (version 0.24.2), a library for machine learning containing tools to tackle classification challenges. Automatic selection of description records and their subsequent expert verification were performed using 14 key words specific to ICH, as well as 64 stop phrases, which, if present in the text, marked the absence of ICH. Selection of texts with the lookup pathology was performed when the following keywords were available in the text (including phrases containing an indication of the hemorrhage type): hemorrhage-, hemato-, hemorrhagic-, intracerebral-, subarachnoid-, epidural-, subdural-, intraventricular-, SAH (subarachnoid hemorrhage), EDH (epidural hemorrhage), SDH (subdural hemorrhage), ICH (intracerebral hemorrhage), IVH (intraventricular hemorrhage), intraparenchymal-. Here, the texts were to have no stop phrases in them: for example, “There are no CT data for intracranial hematoma and brain contusion”, “no evidence of intracranial hemorrhage”, etc. Description of visual representation of any blood, including postoperative or posttraumatic blood, was also considered as presence of the lookup pathology. The description of the hemorrhage included an indication of the contents density from 40 to 90 Hounsfield units (HU). For example, the following description was considered containing the lookup pathology: “The series of CT scans of the left temporal area has hemorrhagic foci up to 20, 11, 8, 6, 4 mm with a density of up to 65 HU. Hemorrhagic contents following the grooves contours are seen in the left parietal area”.

The selection resulted in a dataset (dataset 1) with two classes of text records: with the ICH description and without it. Full texts of X-ray reports were used (containing both a description and a conclusion); the text length ranged from 310 to 3554 characters with spaces. For additional details about the selection algorithm one can refer to our earlier study [15].

To evaluate the model performance, the records from dataset 1 were randomly divided into samples of 7:3, as this is the ratio of the training/test dataset that allows to obtain the optimal metrics for the algorithm quality [16]. Of 3980 records, 2786 were included into the training dataset, 1194 — to the test dataset. Of 1194 test sets, 927 did not contain reference to ICH, 267 had such a reference. All records had a unique identifier, which allowed to exclude data leakage from the training set to the test set.

Seven machine learning algorithms and three text vectorization techniques were selected as models for binary classification. The following algorithms were used: logistic regression, random forest, gradient boosting library (CatBoost, version 1.1.1), support vector machines (SVM), k-nearest neighbors (KNN), gradient boosting library (XGBoost, version 1.7.1) from the Scikit-learn library in Python (version 3.9.7). Each algorithm was searched for optimal hyperparameters by means of the brute-force technique.

In addition to machine learning algorithms from the Scikit-learn library, the authors used the following techniques for vector representation of text records in natural language: bag of words, TF-IDF, and Word2Vec.

The bag of words vectorization technique creates a table (dictionary), in which every unique word in the text is represented by a separate column, and the rows correspond to sentences. If the word is used in a sentence, the table cell contains 1; if the word is not used — 0. TF-IDF estimates the word value for a line and text in general based on a word occurrence in the line. From the mathematical point of view, TF-IDF uses the following formula for determination:

TF-IDF=TF⋅IDF,

where TF is the word occurrence in the line, IDF is the inverse document frequency (the number of times a word occurs in the dataset).

Word2Vec is neural network that can estimate the cosine proximity of word vectors.

The MedRuBertTiny2 version [17] was taken as the pre-trained BERT model. It was tried and tested on the basis of a specifically collected dataset of more than 30,000 medical histories in Russian. This model was created as part of a project on development of a technique for typos correction in patient records using the BERT models to rank candidates (i.e., they were given a score or weight to determine the most relevant and having the larger value to a particular task). MedRuBertTiny2 was additionally trained with the following technical parameters: learning speed — lr=1e-5, n_splits=4, epoch=10.

To additionally train and retest the ensemble of algorithms and the BERT model, a new, independent labeled dataset (dataset 2) was used; it was collected similar to dataset 1, but on a larger number of texts. This set contained 9393 description records (5443 without a pathology description and 3950 with the ICH description), which were divided into training (6790) and test (2603) sets. The texts in datasets 1 and 2 are not repeated.

The performance of the algorithms was assessed using the classification_report function. Mc Nemar’s test was used for statistical analysis. We tested the null hypothesis of the absence of statistically significant differences between the sensitivity and specificity indicators of machine learning algorithms and their ensembles and compared it with the alternative hypothesis of the presence thereof.

To improve the model quality, texts were pre-processed, i.e., all letters in words were converted to lower case (A→a), unnecessary symbols and words (prepositions, conjunctions, particles) were removed, the text was lemmatized and divided into tokens (sentences were divided into combining words). Then, the preprocessed text was vectorized using three techniques: bag of words, TF-IDF, and word2Vec.

## Results

All seven studied machine learning algorithms from the Scikit-learn library were applied to the preprocessed and vectorized text. Each of the machine learning algorithms was tested using all three text vectorization techniques in sequence. The test results are shown in Tables 1–3.

**Table 1:** Results of testing machine learning algorithms using the bag of words text vectorization technique

Algorithm	Accuracy	Completeness	F1-score	Sensitivity	Specificity
***Decision tree***					
Hemorrhage	0.78	0.74	0.76	0.93	0.77
Reference	0.93	0.95	0.94		
***Logistic regression***					
Hemorrhage	0.80	0.85	0.82	0.95	0.85
Reference	0.96	0.95	0.95		
***Random forest***					
Hemorrhage	0.86	0.13	0.22	0.99	0.13
Reference	0.82	0.99	0.90		
***Nearest neighbors***					
Hemorrhage	0.63	0.86	0.73	0.87	0.86
Reference	0.96	0.87	0.92		
***CatBoost***					
Hemorrhage	0.76	0.78	0.77	0.94	0.78
Reference	0.94	0.94	0.94		
***XGBoost***					
Hemorrhage	0.86	0.79	0.83	0.79	0.97
Reference	0.95	0.97	0.96		
***Support vector machines***					
Hemorrhage	0.80	0.86	0.83	0.94	0.86
Reference	0.96	0.94	0.95		

**Table 2:** Results of testing machine learning algorithms using the TF-IDF text vectorization technique

Algorithm	Accuracy	Completeness	F1-score	Sensitivity	Specificity
***Decision tree***					
Hemorrhage	0.67	0.69	0.68	0.81	0.65
Reference	0.91	0.90	0.90		
***Logistic regression***					
Hemorrhage	0.87	0.78	0.82	0.96	0.78
Reference	0.94	0.96	0.95		
***Random forest***					
Hemorrhage	0.88	0.50	0.64	0.98	0.50
Reference	0.87	0.98	0.92		
***Nearest neighbors***					
Hemorrhage	0.77	0.76	0.77	0.93	0.76
Reference	0.93	0.93	0.93		
***CatBoost***					
Hemorrhage	0.82	0.79	0.81	0.94	0.78
Reference	0.94	0.94	0.94		
***Support vector machines***					
Hemorrhage	0.84	0.82	0.83	0.50	0.82
Reference	0.95	0.95	0.95		
***XGBoost***					
Hemorrhage	0.94	0.95	0.94	0.79	0.95
Reference	0.82	0.79	0.80		

**Table 3:** Results of testing machine learning algorithms using the Word2Vec text vectorization technique

Algorithm	Accuracy	Completeness	F1-score	Sensitivity	Specificity
***Decision tree***					
Hemorrhage	0.80	0.59	0.68	0.95	0.59
Reference	0.88	0.95	0.91		
***Logistic regression***					
Hemorrhage	0.81	0.69	0.75	0.95	0.69
Reference	0.91	0.95	0.93		
***Random forest***					
Hemorrhage	0.86	0.76	0.81	0.96	0.76
Reference	0.93	0.96	0.94		
***Nearest neighbors***					
Hemorrhage	0.86	0.77	0.81	0.96	0.77
Reference	0.93	0.96	0.94		
***CatBoost***					
Hemorrhage	0.79	0.69	0.73	0.94	0.78
Reference	0.90	0.94	0.92		
***Support vector machines***					
Hemorrhage	0.81	0.73	0.77	0.95	0.73
Reference	0.92	0.95	0.93		
***XGBoost***					
Hemorrhage	0.92	0.92	0.92	0.73	0.92
Reference	0.71	0.73	0.72		

Analysis of the obtained metrics resulted in the decision to use the stacking technique that included algorithms with the highest metrics, in which training was conducted on two models and the result was transferred to the input of the third. Training and testing were performed on dataset 1 using three text vectorization techniques one-by-one. The results are demonstrated in Table 4.

**Table 4:** Results of testing machine learning algorithm ensembles using three text vectorization techniques

Algorithm	Accuracy	Completeness	F1-score	Sensitivity	Specificity
***Stacking CatBoost, Random LogReg & KNN, TF-IDF***					
Hemorrhage	0.82	0.84	0.83	0.94	0.84
Reference	0.95	0.94	0.95		
***Stacking CatBoost, Random LogReg & KNN, Word2Vec***					
Hemorrhage	0.42	0.14	0.21	0.94	0.14
Reference	0.78	0.94	0.84		
***Stacking CatBoost, Random LogReg & KNN, bag of words***					
Hemorrhage	0.78	0.90	0.84	0.93	0.90
Reference	0.97	0.93	0.95		

Based on the data of Table 4, one can note that the ensemble of machine learning algorithms, consisting of stacking CatBoost, logistical regression, and k-nearest neighbors with the bag of words text vectorization technique (Stacking CatBoost, Random LogReg & KNN, bag of words), had the best results in terms of specificity (p<0.05), while sensitivity indicators in all three techniques of text vectorization did not differ statistically significantly (p>0.05).

**Figure 1.**
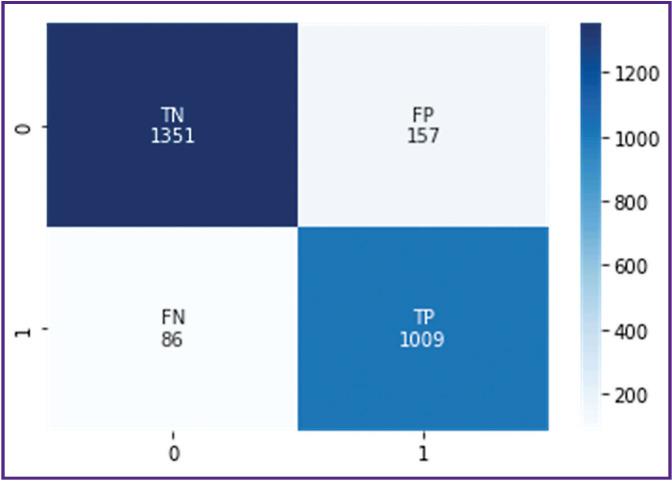
Error matrix for the ensemble of the following algorithms: CatBoost, Random LogReg & KNN, bag of words Vertically, true score of the examination: *0* — examination without signs of intracerebral hemorrhage (true negative result); *1* — examination with signs of hemorrhages (true positive result). Horizontally, ensemble score: *0* — pathology presence was identified incorrectly (false positive result), *1* — pathology absence was indicated incorrectly (false negative result)

This ensemble was additionally trained and tested on dataset 2. The sensitivity was 0.92, the specificity — 0.90. One should note that the metrics did not change significantly (p>0.05). At that, the learning curve shows a slowdown in progress and a plateau, which indicates that a specific limit was reached for such an approach. Figure 1 shows its error matrix with the number of true and false positives and negatives.

**Figure 2.**
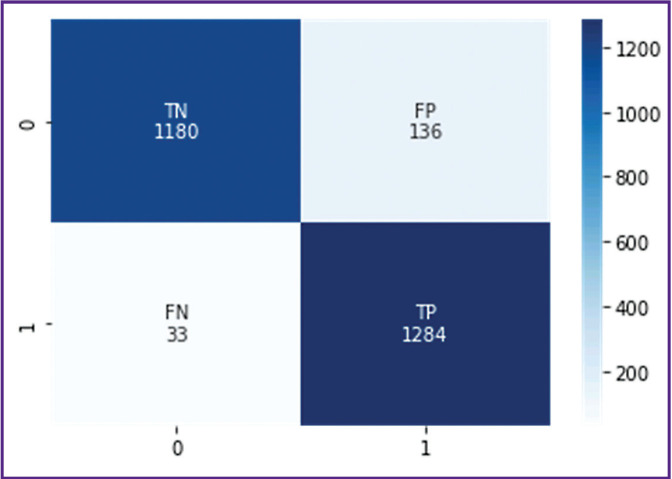
Error matrix for the BERT model Vertically, true score of the examination: *0* — examination without signs of intracerebral hemorrhage (true negative result); *1* — examination with signs of hemorrhages (true positive result). Horizontally, the BERT score: *0* — pathology presence was identified incorrectly (false positive result), *1* — pathology absence was indicated incorrectly (false negative result)

The pre-trained BERT medical model was also additionally trained and tested on the same independent dataset (dataset 2). Sensitivity was 0.97, specificity — 0.90. These metrics are statistically significantly better (p<0.05) compared to the metrics resulted from the additional training and testing of the ensemble of machine learning algorithms on the same dataset, despite the fact that the BERT model was trained on a smaller number of diagnostic X-ray texts. Figure 2 shows its error matrix with the number of true and false positives and negatives.

## Discussion

In the earlier study [15], we discussed the possibility of using a decision tree algorithm for binary classification of brain CT reports to identify ICH. This algorithm has the highest interpretability (compared to other machine learning techniques) combined with simplicity and the possibility of automatic learning [18]. This was the reason for choosing this algorithm at the first, pilot stage to create a program for automatic analysis of diagnostic texts. However, the study revealed that it has significant limitations such as the following: false positives, difficulties with the classification of texts with major variations in description of the presence and absence of the lookup pathology, and the need for manual review of examinations to ensure quality control [15].

For this reason, it was decided to complicate the classifier. This was approached by creating ensembles from several machine learning algorithms and application of several text vectorization techniques that transfer written speech into a format for automatic processing. The trained ensemble showed fairly high results as to the accuracy of operation during the analysis of text descriptions of brain CT scans having traces of intracranial hemorrhages. However, even in this case, quality control required manual revision.

Based on the manual review of an array of description records, which were interpreted automatically and incorrectly, the authors believe that the main reason for the errors is related to the fact that the ensemble of machine learning algorithms does not take into account the semantic peculiarities of the X-ray record structure and the contextual proximity of the terms from the records. For example, the following description record was erroneously labeled as containing the lookup pathological changes in the brain:

“CT scan does not reveal pathological foci of injuries in the brain. In the left parietal and occipital areas, subdural hematoma and pneumocephalus are not detected. In the basal parts of the frontal lobes, SAH is not clearly identified. No other dynamics. The midline structures are not displaced. The lateral ventricles are symmetrical, the contents are homogeneous. The cisterns of the basal brain can be traced and are not deformed. The fissures of the subarachnoid spaces and convexital grooves are not widened. <…> Positive dynamics is seen when compared with the CT scan dated 27 December 2022: the focus of the injury and SAH in the basal frontal areas on the left, pneumocephalus and lamellar subdural hematoma are regressed. Fracture of the left temporal bone. Fracture of the occipital bone. Pathological contents in the cells of the left mastoid process. Polysinusitis”.

The record informs a specialist that pathological changes, such as the injury focus, subdural hematoma, and SAH, regressed and are no longer detected on the brain CT scan; thus, from the point of view of ICH this examination can be interpreted as compliant with the “reference”. However, it is difficult to analyze its description using keywords and stop structures that machine learning algorithms take into account.

Moreover, the authors faced incorrect interpretations in the form of false negative responses. For example, the record was marked out by the ensemble as follows:

“In the basal ganglia and in the left insular lobe switching to the basal parts of the temporal area, a hypodense area with a density of +16...+19 HU and dimension of 50x28x35 mm is detected having a pinpoint hyperdense area up to 5 mm in diameter slightly to the cranial direction from this area in the frontal area. Reduced differentiation of gray and white substance, smoothing of the grooves in the left fronto-parietaltemporal area. ASPECTS in the territory of the left MCA totaled 5 points. <…> No recent bone injury changes were reliably revealed. Conclusion: early CT signs of ischemia in the left fronto-parietal-temporal region. Subacute ischemia in the basal ganglia, insula, and left temporal area. Hyperdense focus in the frontal area on the left — pinpoint hemorrhage? hyperdense vessel? CT control in dynamics is recommended”.

Based on this conclusion, one can assume that the medical officer described a hyperdense area, but was not sure of its substrate. However, it may be a hemorrhage, and erroneous exclusion of this record, depending on the purpose, would be undesirable.

It should be noted that the such inaccuracies could be a result of preprocessing of the dataset. This fact is one of the limitations of this study and requires additional research.

The BERT transformer model, which was additionally trained on a set of diagnostic texts, demonstrated higher accuracy metrics, as it received specific semantic and contextual connections characteristic of X-ray description records. Additional tuning of the model’s hyperparameters and its targeted additional training on datasets with a larger number of description records can further improve its performance, while it seems that additional training of the ensemble of machine learning algorithms within the framework hereof may not lead to a significant result improvement [19].

The tools described herein may work worse on the records of medical officers who describe X-ray images differently from the standard of medical organizations of the Moscow Department of Healthcare or on the records containing grammatical errors. This aspect is also a limitation and requires additional research (possibly using a set of texts from other medical institutions).

Currently, there are many reports on adapting the BERT model to analyze medical texts presented in various languages: Arabic [20], German [21], Turkish [22], Korean [23], Chinese [24], and etc. It is also reported that in order to achieve maximum accuracy in medical NLP tasks traditional machine learning approaches as primary text classification can be combined with BERT for more accurate analysis identifying the lookup features or meanings in texts [25].

The urge to achieve the highest accuracy rates of algorithms to analyze unstructured medical texts is imposed by current problems and limitations generally typical of the AI application in medicine. First of all, these include the quality of data used for training, for example, computer vision algorithms. Creation of high-quality datasets is a time- and labor-consuming process. Unstructured medical texts used to select diagnostic images may contain errors, inconsistencies and missing data, and this will ultimately affect the results accuracy [26]. The more high-capacity automatic selection tools available to medical officers and experts to create such datasets, the better.

Moreover, such tools can be of critical importance to healthcare organizations. For example, they can simplify making various statistical reports and help monitor the operation of medical information systems designed to automate diagnostic, treatment, administrative, support, and other processes [27].

## Conclusion

The trained ensemble of machine learning algorithms demonstrated high performance results in the analysis of text descriptions of the brain CT records with signs of intracranial hemorrhage and, in general, can be used for binary classification. However, manual revision cannot be avoided for the quality control sake. The pre-trained BERT medical transformer model after the additional training on the same dataset demonstrated statistically significantly higher accuracy metrics, which may become even higher with further selection of hyperparameters and additional training of the model on a larger number of diagnostic texts. This evidences the model’s high potential and ways for further improvement in analysis of unstructured medical information in order to identify specific values: for example, the fact of surgical intervention or hemorrhages at different stages of development.

However, the most effective tool to analyze diagnostic text records can result from combining two approaches: an ensemble of machine learning algorithms for primary binary classification and a trained BERT model for in-depth semantic analysis of the text and looking for specific clinical signs in it (for example, to select CT scans with different causes of hemorrhage or at different stages of hemorrhage).
